# Systemic mastocytosis: a rare cause of osteoporosis

**DOI:** 10.11604/pamj.2019.32.169.16640

**Published:** 2019-04-09

**Authors:** Vishnu Vardhan Garla, Kanooz Ul Qadir Chaudhary, Abid Yaqub

**Affiliations:** 1 Division of Endocrinology, Department of Internal Medicine, University of Mississippi Medical Center, Jackson, United State of America; 2 Department of Family Medicine, Holzer Health System, Gallipollis, United State of America; 3 Division of Endocrinology, Department of Internal Medicine, University of Cincinnati, Cincinnati, United State of America

**Keywords:** Osteoporosis, systemic mastocytosis, urticaria, secondary osteoporosis, tryptase, bisphosphonate

## Abstract

A 61-year old female patient who was referred to the endocrine clinic for evaluation of an elevated alkaline phosphatase. She was originally referred to gastroenterology (GI), however no GI causes of elevated alkaline phosphatase was found. Upon fractionation, it was noted that she had elevation in bone specific alkaline phosphatase. Past history was significant for hypertension, atrial fibrillation and menopause 6 years ago. She was also noted to have multiple drug allergies manifesting as urticaria and flushing. Review of the past records revealed a persistently elevated alkaline phosphatase over the last two years. She had no history of falls or fractures. Computed tomography (CT) abdomen done to rule out biliary pathology, revealed osteosclerotic and osteolytic lesion in the pelvis concerning neoplastic disease. Bone marrow biopsy however, was negative for cancer but consistent with systemic mastocytosis (SM). Dual Energy X-ray absorbimetery (DEXA) scan revealed osteoporosis Serum tryptase levels were elevated; further genetic analysis showed a positive CKIT D816 mutation. She was started on bisphosphonates (initially alendronate and then ibandronate). Upon follow up at two years she had not experienced any fractures and her bone mineral density also had improved significantly.

## Introduction

Osteoporosis is defined as a disease characterized by low bone mass and microarchitectural deterioration of bone tissue leading to enhanced bone fragility and a consequent increase in fracture risk [1]. Secondary osteoporosis is defined as osteoporosis which occurs secondary to a disease state or medication [2]. About 30% of postmenopausal women and 50-80% of men have factors contributing to osteoporosis. Systemic mastocytosis is a rare cause of secondary osteoporosis however it may be underdiagnosed due to a lack of awareness [3, 4]. Moreover, treatment of secondary factors of osteoporosis may affect the response to antiresorptive medications [5]. Physicians should consider SM in patients with unexplained osteoporosis and symptoms of mast cell release (flushing, urticarial, diarrhoea, and angioedema) [6]. Prompt diagnosis of this disorder may prevent future fragility fractures and decrease mortality and morbidity.

## Patient and observation

**Figure 1:**
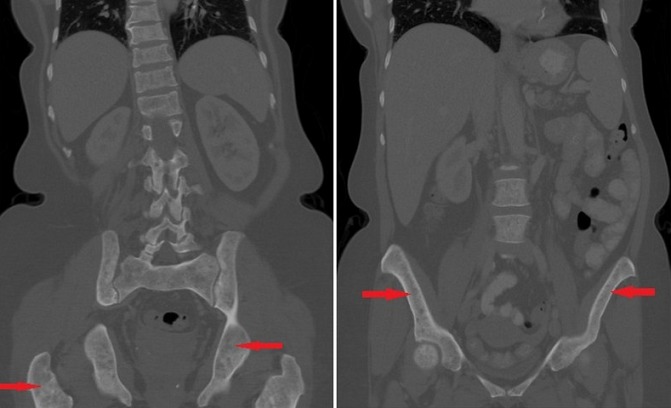
computed Tomography (CT) of the abdomen and pelvis showing osteosclerosis of pelvic bones

**Figure 2:**
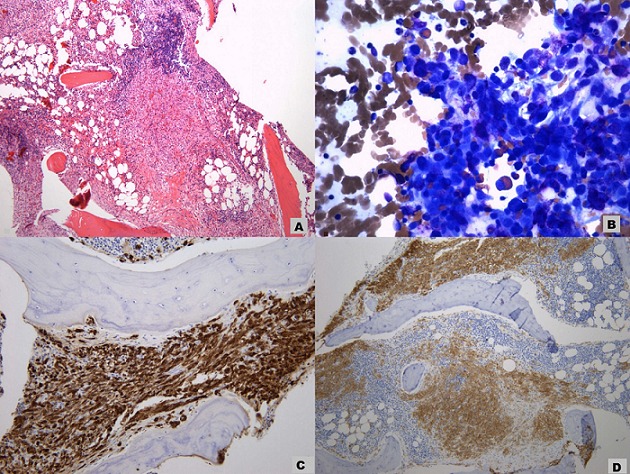
A) paratrabecular mast cell inifltrates occupying the bone marrow; B) neoplastic spindle cell infiltrate in the bone marrow; C) positive staining to tryptase confirming mast cell lineage; D) positive staining to CD 25 consistent with neoplastic mast cells

A 61-year old Caucasian female who was referred to the endocrine clinic for evaluation of elevated serum alkaline phosphatase. She was referred to GI, however, no GI cause of elevated alkaline phosphatase was found. Fractionation of alkaline phosphatase showed elevation in bone specific alkaline phosphatase. She had a past medical history significant for hypertension, atrial fibrillation and menopause 6 years ago. She was also noted to have allergies to multiple medications, the reactions manifesting as urticaria and flushing. She also complained of diffuse pain in her back and extremities. Family history was significant for maternal osteoporosis. Upon review of her medical records, it was noted that her alkaline phosphatase was persistently elevated over the last two years. Ultrasound (USG) of abdomen to evaluate gall bladder disease showed a heterogeneous appearance of the liver, but no gall bladder disease. CT abdomen did not reveal any abnormalities of the liver, however it did show diffuse sclerosis of the pelvis (Figure 1). As this was concerning for bone neoplasia, a positron emission tomography (PET) scan was done which did not reveal any abnormal uptake. Laboratory assessment showed an elevated bone specific alkaline phosphatase, urinary N telopeptide along with a normal parathyroid hormone (PTH) and vitamin D, indicating increased resorption of the bone (Table 1). DEXA scan revealed marked discrepancy between bone mineral density in the femoral neck and lumbar spine versus the distal forearm. T-score at the distal forearm was consistent with the diagnosis of osteoporosis (Table 2). Bone marrow biopsy revealed multifocal dense mast cell masses with predominantly spindle cell morphology most consistent with indolent SM. Immunohistochemical staining was positive for tryptase and CD25 (Figure 2). Genetic testing revealed a positive D816 mutation. She was treated with doxepin and ranitidine for her flushing and urticaria. For osteoporosis, antiresorptive therapy with alendronate 70 mg weekly was initiated, this was later changed to ibandronate 150 mg monthly upon patient's request. Follow up bone densitometry 2 years later showed marked improvement in bone mineral density (BMD) at the lumbar spine. She also experienced resolution of her urticaria and flushing.

**Table 1:** laboratory assessment at presentation

S.No.	Test	Result	Normal value
1.	Bone alkaline phosphatase	36.1	0-21.3 mcg/lt
2.	Alkaline phosphatase isoforms	155	25-165 IU/lt
	Liver fraction	28	26-86%
	Bone fraction	72	11-68%
	Intestinal fraction	0	0-16%
3.	25 (OH) Vitamin D	48.9	32-100 ng/ml
4.	Parathyroid, intact	50.5	8-97 pg/ml
5.	Urinary N-telopeptide	2692	19-63 nmol BCE
6.	N-telopeptide/creatnine ratio	65	5-65 nM BCE/mM Cr
7.	Serum tryptase	158	(1-11.5 mcg/lt)

mcg=micro grams; lt=litre; IU=international units; ng=nanograms; ml=milliliter; pg=pictograms; nmol BCE= nanomoles bone collagen equivalent; mMCr= millimoles of creatinine

**Table 2:** comparison of bone mineral density at diagnosis and two years later

	At presentation		2 years later	
	Bone mineral density (grams/square cm)	T score	Bone mineral density	T score
Lumbar spine	0.922	-2	1.154	-0.2
Left femoral neck	1.099	0.4	1.259	1.6
Left distal radius	0.539	-3.8	0.542	-3.8

## Discussion

Mastocytosis refers to a group of disorders characterized by excessive mast cell accumulation in one or more tissues. It is subdivided into cutaneous mastocytosis when it is limited only to the skin and SM when it infiltrates extra-cutaneous organs [7]. Cutaneous mastocytosis was first described in 1869 by Nettleship. It is the most common form in childhood and often resolves by itself [8]. SM was first described in 1933 by Touraine. It is more common in adults and is characterized by involvement of at least 1 extra-cutaneous organ. The most commonly involved organs are bone, gastrointestinal tract, spleen, and lymph nodes. SM has been further classified into indolent SM, SM with an associated hematological non mast cell disorder (SM-AHNMD), aggressive mastocytosis (ASM) and mast cell leukemia (MCL). The most common form of SM is indolent SM [9]. Clinical features are variable and depend on the system involved, effects of the mediators and the mast cell burden. Cutaneous involvement is very common and seen in over 90% cases. It manifests most commonly as urticaria, flushing, and angioedema. Our patient had urticaria and flushing for many years, we suspect that these were wrongly attributed as allergic reactions to medications. Involvement of the gastrointestinal tract leads to nausea, vomiting, diarrhea, and abdominal pain. Rarely hepatopathy, portal hypertension ascites, hypersplenism, and malabsorption may also be seen [10, 11].

Skeletal involvement occurs in 70% of patients with SM [10]. Van der Veer *et al.* studied 157 patients with indolent SM and found a high prevalence of osteoporotic fractures (38%). Male preponderance was noted, with 62% of the men having osteoporotic manifestations (BMD of < -2.5 or osteoporotic fracture). Older age, men and elevated urinary methyl histamine levels were independently linked to osteoporotic fractures [12]. Bone pain secondary to bone marrow involvement is the hallmark of SM. SM should be strongly considered in any patient presenting with debilitating bone pain and unexplained osteoporosis. The most common site of involvement is the lumbar spine, indicating the preferential involvement of trabecular bone. This could be due to the tendency of the mast cells to colonize the bone marrow [13]. Radiologically, bone involvement is most commonly characterized by focal osteosclerosis and osteolytic bone lesions affecting the axial skeleton as well as the long bones. This may be confused with Pagets disease, lymphoma or diffuse metastatic disease as seen in our patient. Rarely multiple or solitary osteolytic lesions are also seen [14]. Osteoporosis in SM could be either due to neoplastic infiltration of mast cells or due to the effect of inflammatory mediators secreted by the mast cells. Histamine, tryptase and heparin can directly activate the osteoclasts as well as activating the RANK-L pathway which in turn promotes bone resorption. Interleukin (IL)-1, IL-6, tumor necrosis factor (TNF)-alpha can activate osteoclasts through the RANK-L pathway, and also inhibit osteoblasts thereby increasing bone resorption and decreasing bone formation. Increased levels of sclerostin and DKK-1 which are inhibitors of the Wnt pathway have been noted in some studies which may decrease bone formation [13]. SM should be suspected in any patient with unexplained osteoporosis with symptoms of mast cell mediator release.

Elevated levels of tryptase re observed in 95% of indolent SM cases, however chronic kidney disease, urticaria, psoriasis, hematological disorders, onchocercosis and ischemic myocardial disease can also increase tryptase levels. Urinary histamine metabolites can be helpful in diagnosis of SM where the tryptase is normal, however these metabolites can be increased in patients with gastric carcinoid and Zollinger Ellison syndrome [13, 15]. The diagnosis can be definitively established by a bone marrow biopsy. Typically multifocal dense cell masses of mast cells are seen, sometimes spindle shaped or atypical mast cells may also be seen [16]. Genetic analysis for D816VKIT is positive in about 78% of indolent SM cases [17]. Diagnosis is based on meeting at least 1 major and 1 minor or three minor WHO criteria as outlined in Table 3. Treatment of SM is multipronged, aimed at, alleviating the symptoms using antihistamines and mast cell stabilizers, using cytoreductive agents to control the mast cell proliferation and anti-resorptive agents to treat the osteoporosis [13]. Since increase in bone resorption through activation of osteoclasts and RANK-L pathway is the main mechanism of causation of osteoporosis in SM, antiresorptive agents are the treatment of choice. Among bisphosphonates zoledronic acid has been shown to increase bone mineral density the most. The improvement is most significant in the lumbar spine as seen in our patient who had a 14% increase in BMD in the lumbar spine over 2 years [18]. Laroche *et al.* studied 8 patients treated with pamidronate and interferon alpha and found that spinal bone mineral density (BMD) increased by 15% in 2 years whereas the 2 patients treated with pamidronate alone had only a 2% increase in BMD. There was no difference in the decline of bone remodeling markers in both groups [19]. Orsolini *et al.* studied the use of denosumab, a RANK-L inhibitor in 4 patients and noted an improvement in BMD (both spine and femur) and a decrease in CTX [20].

**Table 3:** WHO diagnostic criteria for SM

**Major Criteria**	Multifocal dense aggregates of 15 or more mast cells as detected with tryptase or other special stains in bone marrow or other extra cutaneous organs.
**Minor criteria**	Atypical morphology or spindle shapes in >25 percent of the mast cells in bone marrow sections, bone marrow aspirate, or other extra cutaneous tissues Mutational analysis of KIT showing a codon 816 mutation (eg, Asp816Val) in bone marrow, blood, or extra cutaneous organs Bone marrow or extra cutaneous mast cells expressing the surface markers CD2, CD25 or both. Baseline serum tryptase levels >20ng/ml. This does not apply to AHNMD

## Conclusion

SM is a rare cause of secondary osteoporosis. It needs to be considered in the differential diagnosis of patients with unexplained osteoporosis and symptoms of mast cell release, unexplained vertebral fractures or osteoporosis with severe bone pain. Elevated tryptase levels although, consistent with SM can also be seen in chronic urticaria, hematological disorders, chronic kidney disease and ischemic myocardial disease. Urinary histamine metabolites may be elevated and are useful in rare cases of SM where tryptase levels are normal. Diagnosis can be definitively established by bone marrow biopsy. Bisphosphonates are the mainstay of treatment of osteoporosis secondary to SM and improve the BMD significantly. RANK-L inhibitors (denosumab) can be an alternative in patients who are intolerant of bisphosphonates.

## Competing interests

The authors declare no competing interests.

## References

[1] No author list (1993). Consensus development conference: diagnosis, prophylaxis and treatment of osteoporosis. Am J Med.

[2] Painter SE, Kleerekoper M, Camacho PM (2006). Secondary osteoporosis: a review of the recent evidence. Endocr Pract.

[3] Harper KD, Weber TJ (1998). Secondary osteoporosis: diagnostic considerations. Endocrinol Metab Clin North Am.

[4] Anne Klibanski, Lucile Adams-Campbell, Tamsen Bassford L, Steven Blair N, Scott Boden D, Kay Dickersin (2001). Osteoporosis prevention, diagnosis, and therapy. JAMA.

[5] Mirza F, Canalis E (2015). Management of endocrine disease: secondary osteoporosis (pathophysiology and management). Eur J Endocrinol.

[6] Valent P, Escribano L, Broesby-Olsen S, Hartmann K, Grattan C, Brockow K (2014). Proposed diagnostic algorithm for patients with suspected mastocytosis: a proposal of the European Competence Network on Mastocytosis. Allergy.

[7] Arock M, Valent P (2010). Pathogenesis, classification and treatment of mastocytosis: state of the art in 2010 and future perspectives. Expert Rev Hematol.

[8] Donker ML, Bakker NA, Jaspers WJ, Verhage AH (2008). Two patients with osteoporosis: initial presentation of systemic mastocytosis. J Bone Miner Metab.

[9] Akin C, Metcalfe DD (2004). Systemic mastocytosis. Annu Rev Med.

[10] Peterson AH (1984). Systemic mastocytosis: case report and literature review. J Natl Med Assoc.

[11] Huang TY, Yam LT, Li CY (1987). Radiological features of systemic mast-cell disease. Br J Radiol.

[12] Van der Veer E, van der Goot W, de Monchy JG, Kluin-Nelemans HC, van Doormaal JJ (2012). High prevalence of fractures and osteoporosis in patients with indolent systemic mastocytosis. Allergy.

[13] Rossini M, Zanotti R, Orsolini G, Tripi G, Viapiana O, Idolazzi L (2016). Prevalence, pathogenesis, and treatment options for mastocytosis-related osteoporosis. Osteoporos Int.

[14] Barer M, Peterson LF, Dahlin DC, Winkelmann RK, Stewart JR (1968). Mastocytosis with osseous lesions resembling metastatic malignant lesions in bone. J Bone Joint Surg Am.

[15] Gerdes S, Kurrat W, Mrowietz U (2009). Serum mast cell tryptase is not a useful marker for disease severity in psoriasis or atopic dermatitis. Br J Dermatol.

[16] Donker ML, Bakker NA, Jaspers WJ, Verhage AH (2008). Two patients with osteoporosis: initial presentation of systemic mastocytosis. J Bone Miner Metab.

[17] Metcalfe DD (2008). Mast cells and mastocytosis. Blood.

[18] Rossini M, Zanotti R, Viapiana O, Tripi G, Idolazzi L, Biondan M (2014). Zoledronic acid in osteoporosis secondary to mastocytosis. Am J Med.

[19] Laroche M, Livideanu C, Paul C, Cantagrel A (2011). Interferon alpha and pamidronate in osteoporosis with fracture secondary to mastocytosis. Am J Med.

[20] Orsolini G, Gavioli I, Tripi G, Viapiana O, Gatti D, Idolazzi L (2017). Denosumab for the Treatment of Mastocytosis-Related Osteoporosis: a Case Series. Calcif Tissue Int.

